# Ninjurin-1 drives atherosclerosis progression via *NF-κB/CXCL-8* activation in endothelial cells

**DOI:** 10.3389/fimmu.2025.1676216

**Published:** 2025-11-07

**Authors:** Zhihong Sun, Wenjuan Ma, Feng Ye, Nana Ren, Ke Shen, Nan Dong

**Affiliations:** 1 Department of Neurology, Shaoxing Central Hospital, Shaoxing, China; 2 The Central Affiliated Hospital, Shaoxing University, Shaoxing, China; 3 Cyrus Tang Medical Institute, Collaborative Innovation Center of Hematology, Soochow University, Suzhou, China

**Keywords:** atherosclerosis, Ninjurin-1, inflammatory, NF-κB, endothelial cells

## Abstract

**Introduction:**

Atherosclerosis represents the leading cause of cardiovascular mortality, with persistent inflammation driving residual risk despite lipid-lowering therapies. While Ninjurin-1 (Ninj1) has been implicated in inflammatory diseases, its endothelial-specific role in atherosclerosis remains unclear.

**Methods:**

We conducted integrated molecular, functional, and histological analyses to characterize Ninj1 expression and function in atherosclerosis. Endothelial Ninj1 silencing was performed to assess its effects on NF-κB signaling, CXCL-8 expression, and ox-LDL-induced endothelial dysfunction. *In vivo*, ApoE-/- mice were treated with the Ninj1 inhibitor mPN12 peptide to evaluate its impact on plaque formation and composition.

**Results:**

Ninj1 silencing in endothelial cells suppressed NF-κB signaling and its key inflammatory mediator CXCL-8, conferring protection against ox-LDL-induced endothelial dysfunction by enhancing proliferation and migration while reducing apoptosis (all p < 0.05). In ApoE^-/-^ mice, pharmacological Ninj1 inhibition with mPN12 peptide significantly attenuated plaque development and lipid accumulation while preserving collagen content.

**Discussion:**

Our results provide the first evidence that endothelial Ninj1 functions as a novel activator of the NF-κB/CXCL-8 axis, establishing its causal role in atherosclerosis and highlighting its potential as a targeted anti-inflammatory therapy.

## 1 Introduction

Cardiovascular diseases remain the leading cause of global mortality, with atherosclerosis as their primary pathological basis (1, 2). Recent epidemiological studies reveal a surprisingly high prevalence of subclinical atherosclerosis in middle-aged populations, challenging traditional views of this disease as solely affecting the elderly (3). Recognizing atherosclerosis as a lipid-driven inflammatory disorder has spurred the development of dual-pathway strategies targeting metabolic and immune components. Current lipid-lowering therapies, including high-intensity statins and PCSK9 inhibitors, reduce but fail to eliminate cardiovascular risk, with approximately 50% residual risk persisting in treated populations (4, 5). This residual risk is increasingly attributed to unresolved vascular inflammation. However, most available anti-inflammatory interventions are associated with systemic immunosuppression and a heightened risk of infection, underscoring the urgent need for safer and more selective anti-inflammatory targets (6).

Ninjurin-1 (Ninj1), a small transmembrane protein initially identified as an adhesion molecule, has recently attracted substantial attention for its critical role in inflammation and inflammatory cell death (7–9). Emerging evidence implicates Ninj1 in various inflammatory conditions including rheumatoid arthritis, asthma, and inflammatory bowel disease (10, 11), with clinical studies now establishing its circulating levels as a biomarker for atherosclerotic stroke risk (12). Particularly noteworthy is its function as a key regulator of plasma membrane rupture during inflammatory cell death, amplifying inflammatory cascades in disease states (13, 14). Animal studies demonstrate that Ninj1 inhibition through genetic knockout or monoclonal antibodies can mitigate tissue damage and inflammation in models of pulmonary fibrosis, multiple sclerosis, and liver injury (15, 16).

The regulatory role of Ninj1 in endothelial function presents a compelling paradox in vascular biology: While Jeon et al. reported that Ninj1 promotes atherosclerosis via macrophage inflammation but lacks functional relevance in aortic endothelial cells (16), earlier studies in diabetic models demonstrate endothelial Ninj1 expression and its pathogenic role (17), suggesting a cell type and disease-specific heterogeneity in Ninj1 biology. Given endothelial dysfunction’s pivotal role as both the initiator and perpetuator of atherosclerosis, resolving this paradox is essential for understanding Ninj1’s potential involvement in vascular inflammation and atherosclerosis. In this study, we investigated the endothelial-specific role of Ninj1 in atherosclerosis through complementary *in vitro* and *in vivo* approaches, and further elucidated the molecular and signaling mechanisms underlying its effects.

## 2 Materials and methods

### 2.1 Ethics statement

The study was approved by the Animal Care and Use Committee of the Central Affiliated Hospital, Shaoxing University. All experimental procedures were performed in accordance with the recommendations of the Guide for the Care and Use of Laboratory Animals, published by the US National Institutes of Health (NIH Publication No. 85–23, revised 1996).

### 2.2 Animals and establishment of atherosclerosis model

Seven-week-old male ApoE^-/-^ mice (C57BL/6J background, n=24) were purchased from Cyagen Biosciences (Suzhou, China) and housed in specific pathogen-free (SPF) conditions at Shaoxing University Animal Research Center, maintained at 23 ± 2°C with 55 ± 5% humidity under a 12-hour light/dark cycle. After one week of acclimatization, mice were randomly divided into four groups (n=6 per group): (a) normal diet (ND) + saline (100 μL, intraperitoneal injection every other day), (b) ND + mPN12 peptide (100 μg in 100 μL saline, i.p., every other day), (c) high-cholesterol diet (HFD) + saline (100 μL, i.p., every other day), and (d) HFD + mPN12 peptide (100 μg in 100 μL saline, i.p., every other day). The HFD (21% fat, 0.15% cholesterol) was provided throughout the 12-week experimental period. The mPN12 peptide was designed to competitively inhibit Ninj1’s adhesion domain (Pro26-Asn37) based on prior structural studies (18).

### 2.3 *In vivo* experiments and analysis of atherosclerotic plaques in mice

At the experimental endpoint, mice were humanely euthanized by intraperitoneal injection of sodium pentobarbital (150 mg/kg) following institutional guidelines. For tissue collection, the chest and abdomen were opened, and the vasculature was perfused with PBS followed by 4% paraformaldehyde (PFA) via left ventricular puncture. The entire aorta from aortic arch to iliac bifurcation was carefully dissected and cleaned of perivascular adipose tissue. Hearts were fixed in 4% PFA overnight for subsequent aortic root analysis. Aortic tissues were processed for either cryopreservation (snap-frozen in liquid nitrogen and stored at -80°C for molecular studies) or histological preparation (embedded in optimal cutting temperature compound for cryosectioning). Serial sections (6μm thick) of the aortic root were cut. Sections were systematically sampled every 5th section (resulting in a 30-μm interval between analyzed sections) and allocated to hematoxylin and eosin (HE), Oil Red O, and Sirius Red staining for histological evaluation of morphology, lipid deposition, and collagen content, respectively. A minimum of 3 sections per staining per animal were analyzed. All histopathological images were captured using a light microscope (Leica) and analyzed with Image-Pro Plus 6.0 software (Media Cybernetics). For quantitative assessments: (a) Plaque area was measured by threshold adjustment of lesion color domains, with plaque burden calculated as (plaque area/vessel area) ×100; (b) Collagen content was quantified by measuring the integrated optical density (IOD) of Sirius Red staining within the plaque area, normalized to the total plaque area (IOD/Area) using Image-Pro Plus software; (c) Lipid deposition was quantified from Oil Red O-stained sections as (lipid area/tissue area ×100).

### 2.4 Immunofluorescence staining of aortic root sections

Cryosections of aortic roots were fixed in cold acetone, blocked with 5% BSA for 30 min, and incubated overnight at 4C with rabbit anti-Ninj1 (1:200, Abcam, ab229288) and rat anti-CD31 (1:200, BD Biosciences). After washing, sections were incubated with Alexa Fluor 488-conjugated anti-rabbit IgG and Alexa Fluor 594-conjugated anti-rat IgG (1:400, Invitrogen) for 1 h at room temperature. Nuclei were counterstained with DAPI. Images were acquired using a Leica fluorescence microscope, and co-localization analysis was performed using ImageJ.

### 2.5 Cell culture and lentiviral transduction

Human umbilical vein endothelial cells (HUVECs) purchased from the Cell Bank of Chinese Academy of Sciences (Shanghai, China) were cultured in endothelial cell-specific medium (ScienCell, USA) supplemented with 5% fetal bovine serum and 1% penicillin/streptomycin at 37°C in a 5% CO2 humidified incubator. For lentiviral transduction, HUVECs (passage 4-6) were seeded in 6-well plates at 1×10^5 cells/well and transduced with either control lentivirus (sh-NC) or Ninj1-targeting lentivirus (sh-Ninj1, Genomeditech, Shanghai) at an MOI of 20 in 300 μL viral supernatant plus 700 μL fresh medium for 12–16 hours, followed by PBS washing and continued culture for 72 hours. The shRNA constructs (four candidates targeting human NINJ1) were designed and synthesized by GenePharma (Suzhou, China) and cloned into the LV3 (H1/GFP&Puro) lentiviral backbone. The sequences are provided in **Supplementary Table S1**. Among the four shRNAs tested, shNINJ1#2 (5′-GGGTGCTGCTCATCTTCCTTG-3′) achieved the highest knockdown efficiency, as validated by RT-qPCR, and was therefore used in subsequent functional assays (**Supplementary Figure S1**).

For quality control, GFP-positive cells were sorted using fluorescence-activated cell sorting (FACS) to establish pure sh-NC and sh-Ninj1 populations, with transduction efficiency exceeding 99.5% (**Supplementary Figure S2**). Functional assays included four experimental conditions: (a) untreated control, (b) 50 μg/mL ox-LDL (Yeasen Biotechnology) alone, (c) ox-LDL + sh-NC, and (d) ox-LDL + sh-Ninj1, with ox-LDL treatment initiated 72 hours post-transduction for 24 hours before subsequent proliferation (CCK-8), migration (scratch wound), and apoptosis assays. All experiments were conducted with cells at 80-90% confluence, and three independent biological replicates were performed for each experimental condition.

### 2.6 RNA extraction, retrotranscription and real-time PCR

Total RNA from human umbilical vein endothelial cells (HUVECs) was extracted using TRIzol reagent (Invitrogen, USA, Cat#15596026) according to the manufacturer’s instructions. cDNA synthesis was performed using the PrimeScript™ RT Reagent Kit (Takara Bio, Japan, Cat#RR037A) with 1 μg of total RNA per reaction. Quantitative real-time PCR was carried out using TB Green^®^ Premix Ex Taq™ II (Takara Bio, Japan, Cat#RR820A) on a QuantStudio 6 Flex Real-Time PCR System (Applied Biosystems, USA). The thermal cycling protocol included an initial denaturation at 95°C for 30 sec, followed by 40 cycles of 95°C for 5 sec and 60°C for 30 sec. Melting curve analysis (60–95°C) confirmed primer specificity. GAPDH and β-actin served as internal controls. For murine tissues, due to the absence of CXCL-8 in the mouse genome, Cxcl1, Cxcl2, and Tnfaip3 were selected as representative NF-κB regulated inflammatory genes for quantitative analysis. Gene-specific primers were designed using PrimerBank and validated for efficiency prior to use. The primer sequences used for RT-qPCR are detailed in **Supplementary Table S2**.

### 2.7 Western blot analysis

Total cellular protein was extracted using RIPA lysis buffer (Beyotime, P0013B) containing 1% PMSF (Beyotime, ST506). Protein concentration was determined using a BCA Protein Assay Kit (Beyotime, P0012). Equal amounts of protein (30 μg) were separated by 10% SDS-PAGE electrophoresis and transferred to PVDF membranes (Millipore, IPVH00010). After blocking with 5% skim milk at room temperature for 1 hour, the membranes were incubated overnight at 4°C with the following primary antibodies: rabbit anti-human Ninj1 (1:1000, Abcam, ab229288), rabbit anti-human CXCL-8 (1:1000, CST, #39765), rabbit anti-human p-NF-κB p65 (1:1000, CST, #3033), rabbit anti-human NF-κB p65(1:1000, CST, #8242), and mouse anti-human β-actin (1:5000, Sigma, A1978). Membranes were washed three times with TBST and incubated with HRP-conjugated secondary antibodies (goat anti-rabbit IgG, 1:5000, Abcam, ab6721) at room temperature for 1 hour. Signals were detected using an enhanced chemiluminescence (ECL) detection system (New Cell & Molecular Biotech, Suzhou, China). Grayscale analysis was performed using Image Lab 6.0 software. GAPDH served as the internal control, and the relative expression of target proteins was calculated as follows: target protein grayscale value/internal reference protein grayscale value.

### 2.8 Cell proliferation assay

Cell proliferation was assessed using the CCK-8 assay (Dojindo Laboratories). Briefly, HUVECs were seeded in 96-well plates (5×10³ cells/well, triplicate wells per group) and cultured under standard conditions. After 24 h of attachment, cells were treated according to experimental groups and incubated with 10% CCK-8 reagent for 2–4 h at 37°C. Absorbance was measured daily at 450 nm (reference 630 nm) using a microplate reader (BioTek) for 7 consecutive days to generate growth curves.

### 2.9 Colony formation and migration assays

For colony formation, single-cell suspensions (500 cells/well) were seeded in 6-well plates and cultured for 10–14 days until visible colonies formed. Colonies were fixed with 4% paraformaldehyde (15 min), stained with 0.1% crystal violet (10 min), and counted (colony formation rate = [colony number/seeded cells] ×100). Cell migration was evaluated using scratch assays: confluent monolayers in 6-well plates were scratched with 200 μL pipette tips, washed with PBS, and cultured in serum-free medium. Wound closure was monitored at 0, 6, 12, and 24 h using an inverted microscope (Leica), with migration distances quantified using ImageJ.

### 2.10 Apoptosis analysis

Apoptosis was detected using Annexin V-FITC/PI staining (BD Biosciences). After treatments, cells (1×10^5^) were collected, washed with cold PBS, and resuspended in 100 μL binding buffer containing 5 μL Annexin V-FITC and 5 μL PI. After 15 min incubation in dark, samples were analyzed by flow cytometry (BD FACSVerse). Early apoptotic (Annexin V^+^/PI^-^) and late apoptotic (Annexin V^+^/PI^+^) populations were quantified.

### 2.11 Data quantification and statistical analysis

Statistical analyses were performed using GraphPad Prism 9.0. Continuous variables are presented as mean ± SEM. Intergroup comparisons used unpaired two-tailed t-tests for two groups or one-way ANOVA with Tukey’s *post-hoc* test for multiple groups. A p-value <0.05 was considered statistically significant. All experiments included ≥3 biological replicates with technical triplicates.

## 3 Results

### 3.1 Ninj1 inhibitory peptide suppresses atherosclerotic plaque formation in ApoE^−/−^ mice

**Figure 1:**
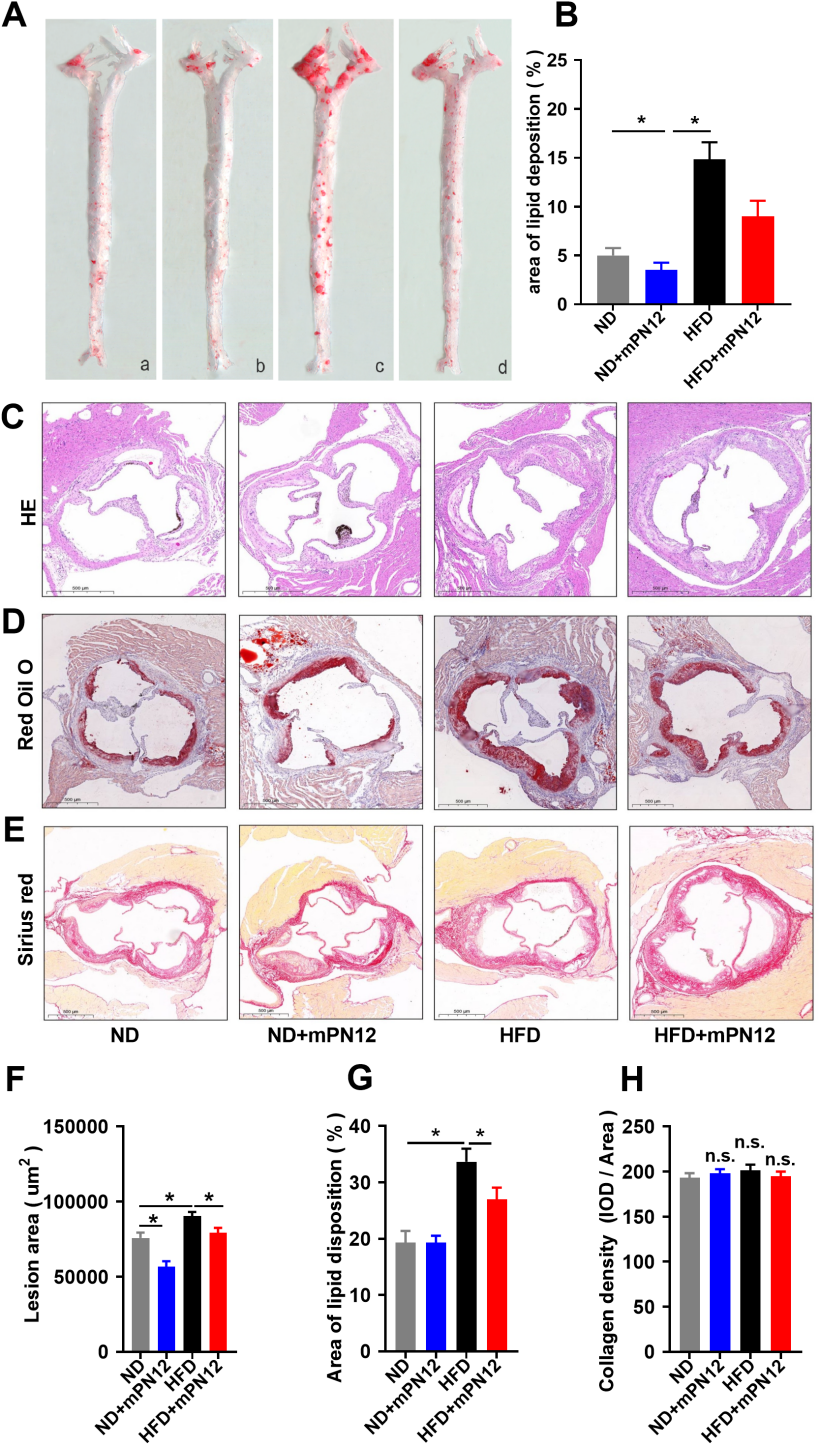
Ninj1 Inhibition Attenuates Atherosclerotic Plaque Formation in ApoE^-^/^-^ Mice. **(A)** Representative images of en face with Oil Red O staining of aortas of ApoE^-/-^ mice with normal-diet (ND) or high-fat diet (HFD) with or without mPN12. Red staining indicates lipid-rich plaques. **(B)** Lesion occupation on the whole aorta was quantified. **(C)** Representative HE staining of aortic root sections. **(D)** Oil Red O staining of plaque lipid content in aortic root sections. **(E)** Sirius Red staining of collagen deposition (red) in aortic root plaques. **(F)** Quantitative analysis of atherosclerotic lesion area in aortic root sections. **(G)** Quantitative analysis of lipid deposition area in aortic root sections. **(H)** Quantitative analysis of collagen content in aortic root plaques. Data are presented as mean ± SEM (n = 6 mice per group), statistical significance was determined by one-way ANOVA followed by Tukey’s *post hoc* test. *p <0.05; n.s., not significant.

To investigate Ninj1’s role in atherosclerosis, we synthesized mPN12, a competitive inhibitory peptide targeting Ninj1’s adhesion domain (Pro26-Asn37) and evaluated its effects on plaque development in ApoE^−/−^ mice. Gross Oil Red O staining of the entire aorta demonstrated that high-fat diet (HFD)-fed mice exhibited significantly increased plaque burden compared to normal diet (ND) controls (16.27 ± 3.23% *vs*. 5.10 ± 1.57%, p=0.001). Notably, mPN12 treatment reduced plaque burden in HFD-fed mice by 42.5% (9.35 ± 3.34% *vs*. 16.27 ± 3.23% in HFD controls, p=0.025) (**Figures 1A, B**). Histological analysis of aortic root sections further revealed that mPN12 reduced plaque area in HFD mice by 8.8% (0.83 ± 0.36 mm² *vs*. 0.91 ± 0.65 mm², p=0.035) (**Figures 1C, F**) and decreased lipid content within plaques (27.3 ± 5.2% *vs*. 33.8 ± 6.7%, p<0.05) (**Figures 1D, G**). However, collagen deposition was not significantly altered in the treatment group compared to the control group (210.28 ± 3.9 *vs*. 195.65 ± 9.78, p=0.132) (**Figures 1E, H**), indicating that mPN12 selectively inhibits early plaque formation without compromising stability.

### 3.2 Ninj1 is predominantly expressed in endothelial cells *in vivo*

**Figure 2:**
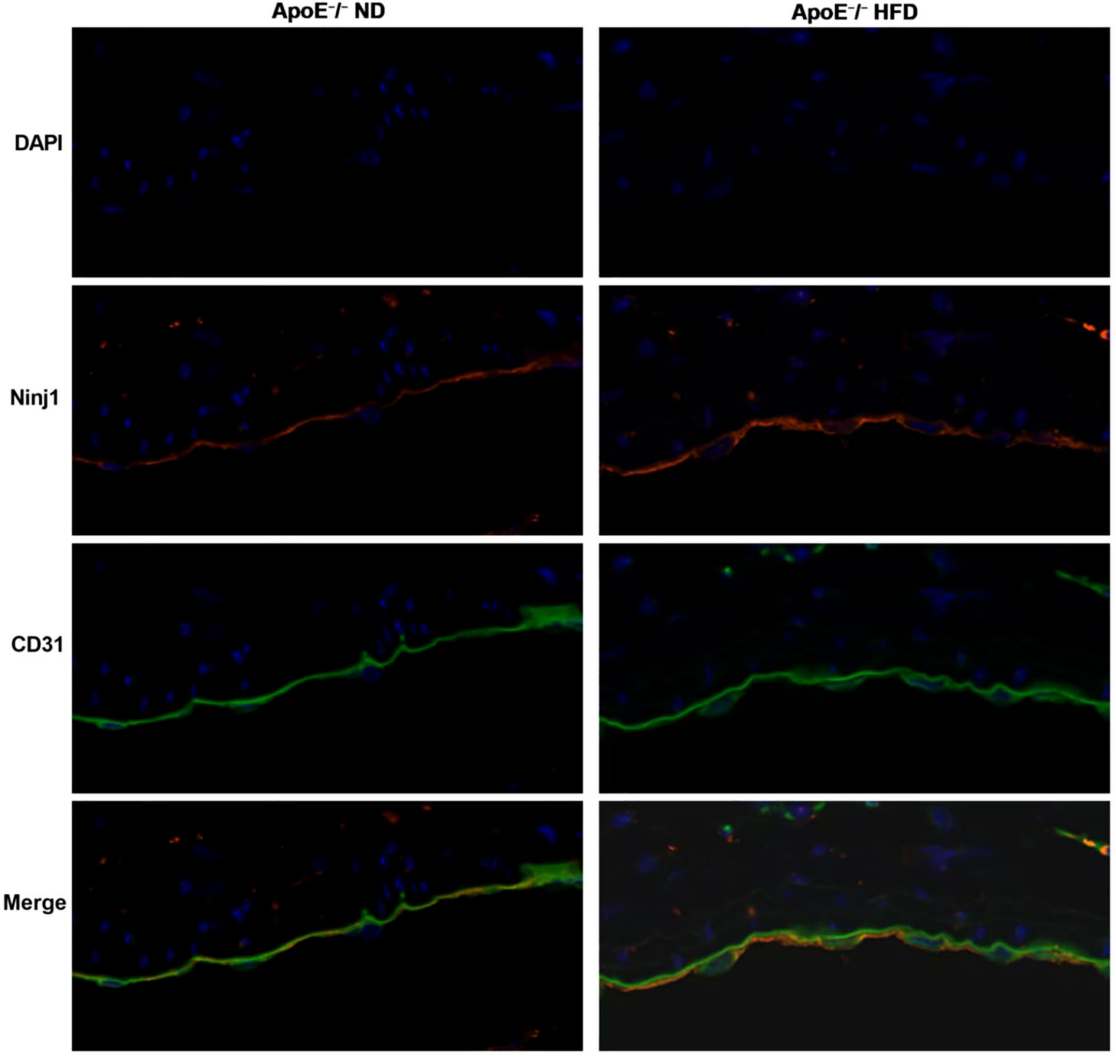
Ninj1 is upregulated and co-localizes with endothelial marker CD31 in aortic roots of ApoE^-^/^-^mice. Representative immunofluorescence images showing Ninj1 (red) and CD31 (green) staining in aortic root sections from ApoE^-^/^-^ mice fed a normal diet (ND) or high-fat diet (HFD). Nuclei were counterstained with DAPI (blue). Ninj1 expression is markedly enhanced and co-localized with CD31^+^endothelial cells under HFD conditions. Scale bar = 20μm.

To confirm the endothelial localization of Ninj1 in atherosclerotic lesions, immunofluorescence co-staining was performed on aortic root sections from ApoE^-^/^-^ mice fed a normal diet (ND) or a high-fat diet (HFD). As shown in **Figure 2**, Ninj1 (red) was mainly localized along the luminal surface and co-localized with the endothelial marker CD31 (green). Notably, Ninj1 expression was markedly increased in CD31^+^ endothelial cells of HFD-fed mice compared with ND controls, providing direct *in vivo* evidence that Ninj1 is predominantly expressed in endothelial cells and upregulated under atherogenic conditions.

### 3.3 Ninj1 modulates endothelial inflammation via CXCL-8/NF-κB signaling pathway

**Figure 3:**
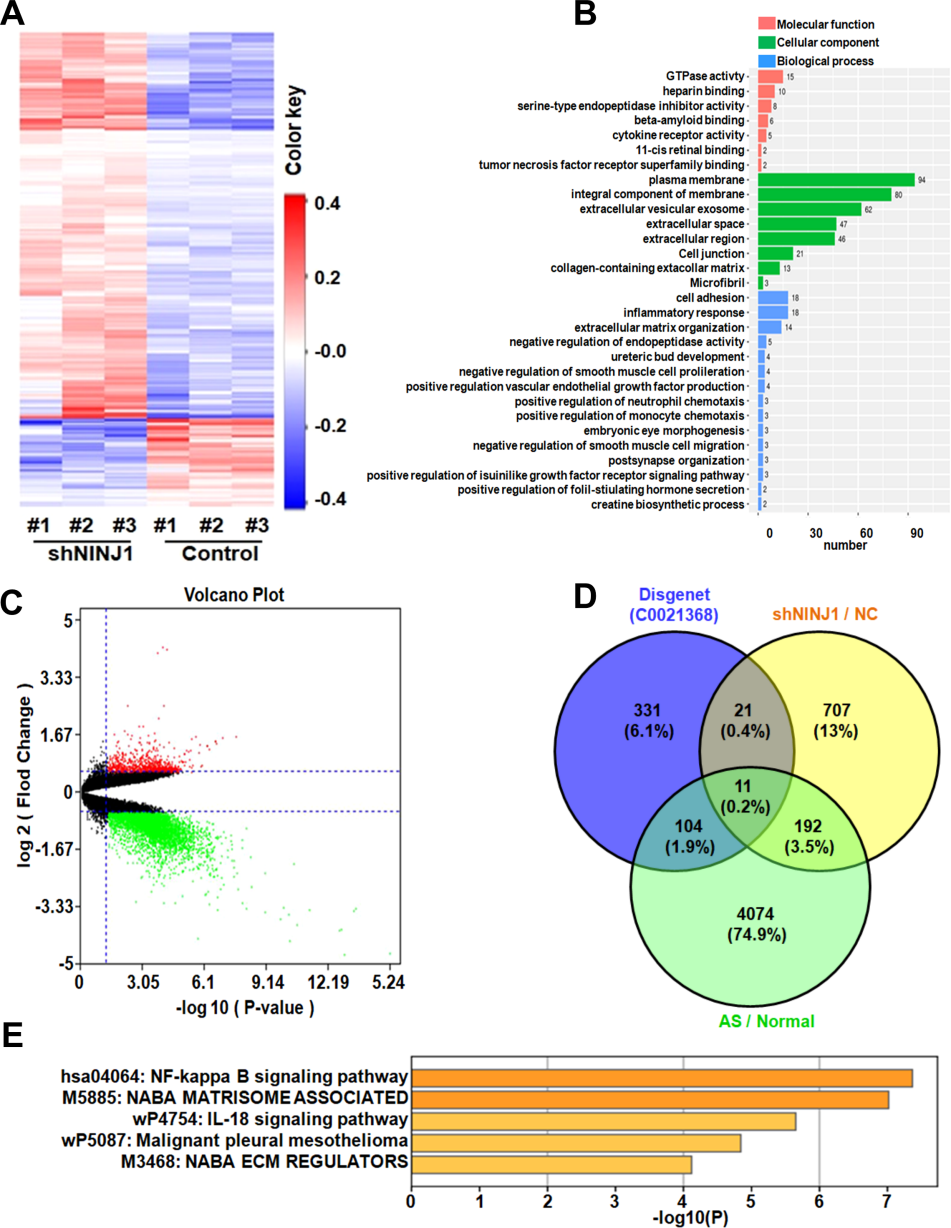
Ninj1 Regulates Atherogenic Pathways through Transcriptional Reprogramming. **(A)** Heatmap of DEGs in Ninj1-silenced HUVECs (red: upregulated; blue: downregulated). **(B)** GO term enrichment categorized by biological process (blue), molecular function (orange), and cellular component (green). **(C)** Volcano plot of DEGs in human atherosclerotic plaques (n=9) *vs*. normal aorta (n=10) (red: log2FC >2, p<0.01). **(D)** Venn diagram intersecting current study DEGs, AS-associated genes (GSE57691), and inflammation-related genes (DisGeNET C0021368), identifying 11 shared genes. **(E)** KEGG pathway enrichment scores (-log10[FDR]) for core genes.

To elucidate the molecular mechanisms by which Ninj1 promotes atherosclerosis, we performed transcriptomic sequencing of Ninj1-silenced HUVECs, identifying 430 differentially expressed genes (DEGs; |log2FC| >1, FDR <0.05), including 350 upregulated and 80 downregulated genes (**Figure 3A**). Hierarchical clustering revealed distinct inflammatory gene expression patterns, and GO enrichment analysis highlighted significant involvement in cell adhesion and inflammatory responses, with cytokine activity representing a key molecular function (**Figure 3B**).

Comparative analysis of the GSE57691 dataset (9 atherosclerotic lesions *vs*. 10 normal aortic tissues) identified 4,381 disease-associated DEGs (log_2_FC >1.5, p<0.05) (**Figure 3C**). Intersection of three datasets: our Ninj1-associated DEGs, atherosclerosis-related genes from GSE57691, and inflammation-associated genes from DisGeNET C0021368, yielded 11 overlapping genes including CXCL-8, TNFAIP3, and CXCL-1 (**Figure 3D**). KEGG pathway analysis confirmed significant enrichment of these core genes in NF-κB signaling, suggesting Ninj1 regulates endothelial inflammation through this pathway in atherosclerosis (**Figure 3E**).

### 3.4 Ninj1 regulates endothelial inflammation via CXCL-8/NF-κB signaling

**Figure 4:**
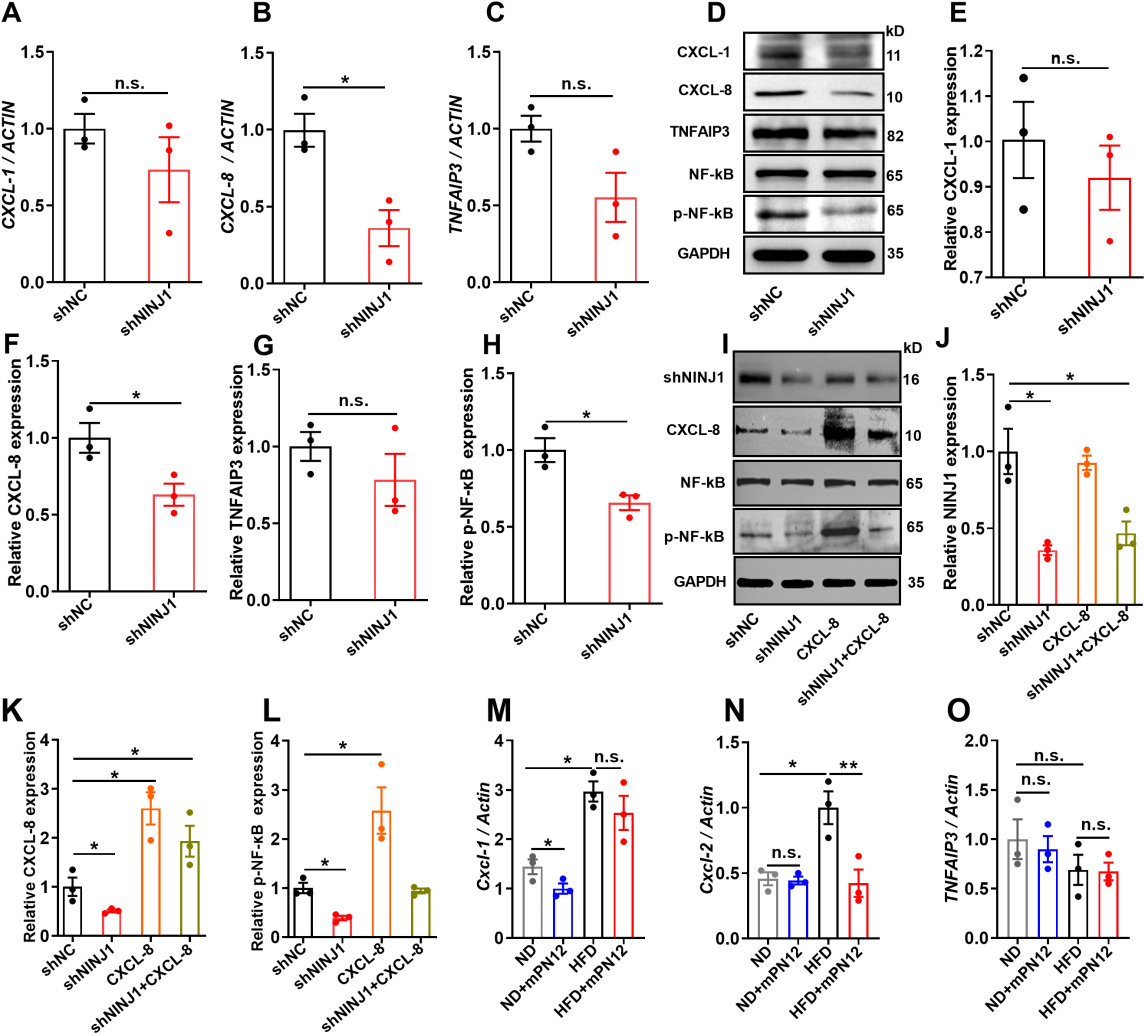
Ninj1 silencing suppressed CXCL-8/NF-κB signaling in endothelial cells and its functional rescue. **(A-C)** mRNA expression levels of CXCL-1, CXCL-8, and TNFAIP3 normalized to ACTIN. **(D)** Representative Western blot showing protein expression of CXCL-1, CXCL-8, TNFAIP3, NF-κB p65, phosphorylated NF-κB p65 (p-NF-κB), with GAPDH as loading control. **(E-H)** Quantitative analysis of protein expression of CXCL-1, CXCL-8, TNFAIP3 and p-NF-κB normalized to GAPDH. **(I-L)** Rescue experiments showing that CXCL-8 overexpression restored NF-κB phosphorylation suppressed by Ninj1 knockdown. Representative blots and quantification are shown. **(M-O)** RT-qPCR analysis of Cxcl1, Cxcl2, and Tnfaip3 mRNA levels in aortic tissues from ApoE^-^/^-^ mice fed a normal diet (ND), ND with mPN12, high-fat diet (HFD), or HFD with mPN12. mPN12 treatment decreased Cxcl2 expression, while Cxcl1 showed a downward trend without significance and Tnfaip3 remained unchanged. Data are presented as mean ± SEM (n=3). Statistical significance was determined by one-way ANOVA followed by Tukey’s *post hoc* test. *p < 0.05, **p < 0.01; n.s., not significant.

To validate the transcriptomic findings, we focused on the NF-κB signaling pathway and its associated genes (CXCL-8, TNFAIP3, and CXCL-1), which are critically involved in inflammatory responses. RT-qPCR analysis revealed that CXCL-8 mRNA expression was significantly downregulated in Ninj1-silenced HUVECs compared to control cells (p=0.016), while TNFAIP3 and CXCL-1 mRNA levels showed a decreasing trend without statistical significance (p>0.05) (**Figures 4A–C**). Further analysis by Western blot demonstrated that CXCL-8 protein expression was markedly reduced in Ninj1 knockdown cells (p=0.037), whereas no significant differences were observed for TNFAIP3 or CXCL-1 protein levels (**Figures 4D–G**). Importantly, Ninj1 silencing significantly suppressed the phosphorylation of NF-κB p65 (p=0.019) (**Figure 4H**), indicating inhibition of NF-κB pathway activation.

To further confirm the causal relationship between CXCL-8 and NF-κB activation, we performed a rescue experiment by overexpressing CXCL-8 in Ninj1-silenced HUVECs. As shown in **Figures 4I–L**, CXCL-8 overexpression restored NF-κB phosphorylation that had been suppressed by Ninj1 knockdown, supporting that CXCL-8 functions as a key downstream effector and positive feedback mediator of the Ninj1/NF-κB signaling axis.

To evaluate the *in vivo* relevance of this pathway, we performed RT-qPCR analysis on RNA extracted from aortic tissues of ApoE^-^/^-^ mice with or without mPN12 treatment. Because mice lack a direct homolog of human CXCL-8, we measured the expression of Cxcl1, Cxcl2, and Tnfaip3, which are functional counterparts involved in NF-κB–mediated inflammatory regulation. As shown in **Figures 4M–O**, mPN12 treatment markedly decreased Cxcl2 expression, whereas Cxcl1 displayed a downward trend that did not reach statistical significance, and Tnfaip3 expression remained unchanged. These findings are consistent with our *in vitro* results, suggesting that pharmacological inhibition of Ninj1 attenuates inflammation at least partly through suppression of the NF-κB/CXCL-8 axis *in vivo*.

### 3.5 Silencing Ninj1 ameliorates ox-LDL-induced endothelial dysfunction

**Figure 5:**
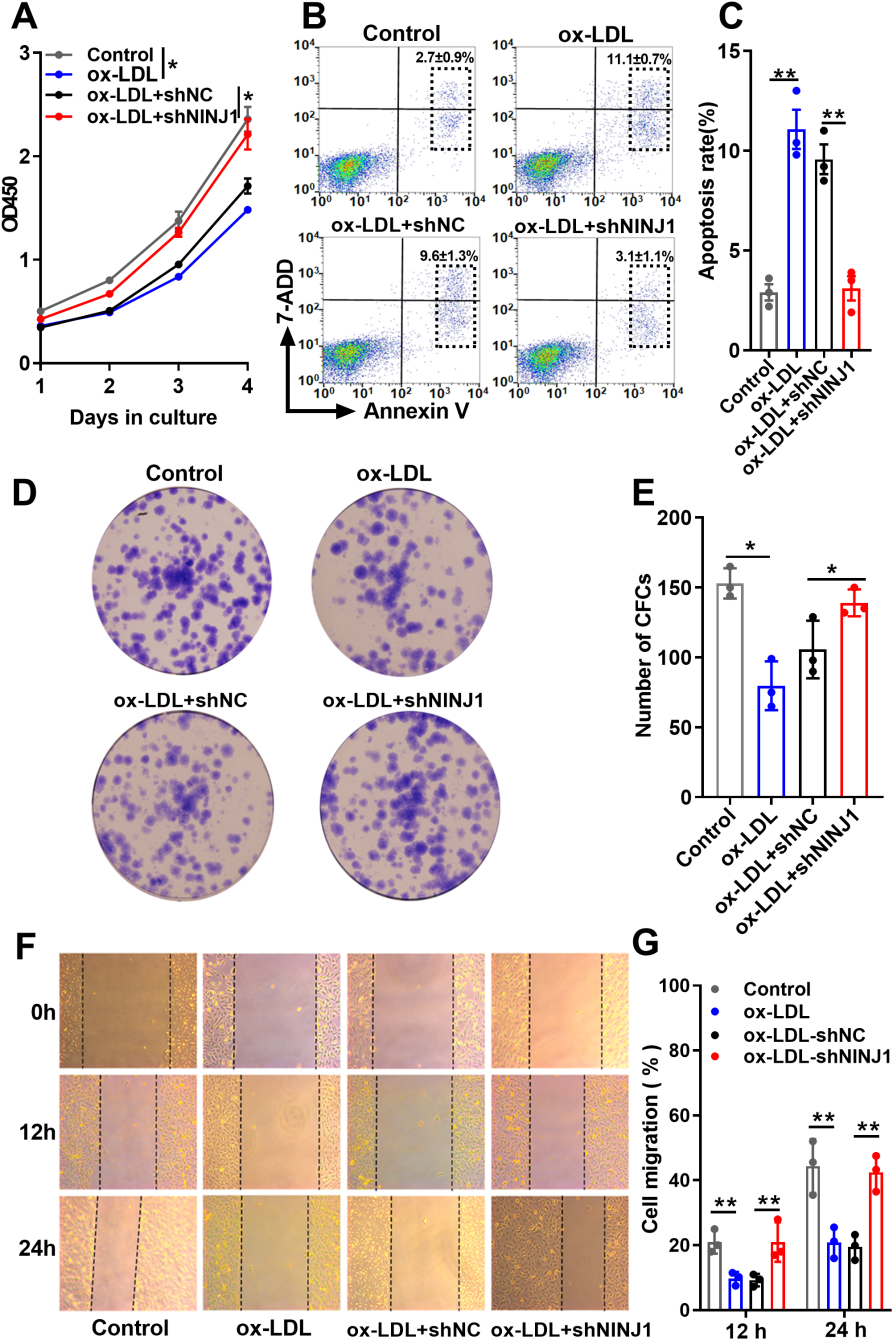
Ninj1 silencing alleviates ox-LDL-induced endothelial cell dysfunction. **(A)** CCK-8 assay showing proliferation curves of HUVECs under different treatments (Control, ox-LDL, ox-LDL+shNC, and ox-LDL+shNINJ1). **(B, C)** Representative flow cytometry plots showing Annexin V-FITC/PI staining and quantification of total apoptosis (sum of early and late apoptotic cells). **(D)** Representative images of colony formation assays. **(E)** Quantitative analysis of colony numbers. **(F)** Representative images of wound-healing migration assays at 0 h, 12 h, and 24 h. **(G)** Quantitative analysis of wound closure percentage. Data are presented as mean ± SEM (n=3). Statistical significance was determined by one-way ANOVA followed by Tukey’s *post hoc* test. *p < 0.05, **p< 0.01.

Given the critical role of endothelial dysfunction in atherosclerosis initiation, we examined whether Ninj1 silencing protects endothelial cells from ox-LDL-induced damage. Functional assays demonstrated that Ninj1 silencing significantly attenuated ox-LDL-induced endothelial damage in HUVECs. CCK-8 proliferation assays revealed that ox-LDL treatment (50 μg/mL, 24 h) markedly reduced cell viability compared to untreated controls (p=0.015), while Ninj1 silencing restored proliferation to baseline levels (**Figure 5A**). This protective effect was confirmed by colony formation assays, where Ninj1-depleted cells exhibited a 64% increase in clonogenic survival compared to ox-LDL-treated controls (31.07 ± 2.32% *vs* 18.93 ± 3.61%, p=0.021) (**Figures 5D, E**). Migration capacity, as assessed by wound healing assays, showed similar recovery, with Ninj1-silenced cells achieving 22.59 ± 5.85% wound closure at 12h versus 9.35 ± 1.94% in ox-LDL-treated controls (p<0.001) (**Figures 5F, G**).

Flow cytometry analysis further demonstrated that Ninj1 knockdown markedly reduced ox-LDL–induced apoptosis in HUVECs. The representative gating strategy and quantitative results are shown in **Figures 5B, C**. Apoptotic rates were calculated as the sum of early and late apoptotic cells. Compared with the ox-LDL and ox-LDL + sh-NC groups, Ninj1 silencing significantly decreased total apoptosis (11.1 ± 1.7% *vs*. 3.1 ± 1.1%, p < 0.001), reaching levels comparable to untreated controls (2.9 ± 0.7%). Together, these findings demonstrate that Ninj1 silencing preserves endothelial homeostasis by maintaining proliferative capacity, migratory function, and cell survival under pro-atherogenic conditions.

## 4 Discussion

This study provides the first comprehensive evidence that endothelial Ninj1 promotes atherosclerosis by activating the NF-κB/CXCL-8 signaling axis. Using both *in vivo* and *in vitro* models, we demonstrate that inhibition of Ninj1 attenuates atherosclerotic plaque formation and lipid deposition. Furthermore, our *in vitro* findings indicate that Ninj1 inhibition preserves endothelial function by enhancing cell survival. Collectively, our *in vivo* and *in vitro* findings reveal a previously underappreciated endothelial-specific mechanism by which Ninj1 contributes to atherosclerosis. Immunofluorescence co-staining of aortic root sections from ApoE^-^/^-^ mice further confirmed that Ninj1 is predominantly localized to CD31^+^ endothelial cells, with markedly increased expression in HFD-fed mice compared with ND-fed controls. These results provide direct histological evidence linking Ninj1 to endothelial cells *in vivo* and support its role as a key mediator of vascular inflammation and early atherosclerosis.

Endothelial dysfunction is widely recognized as a key initiating event in atherosclerosis (19). We observed that Ninj1 knockdown significantly mitigated ox-LDL-induced endothelial injury by restoring proliferative and migratory capacities and reducing apoptosis. Mechanistically, this protective effect was mediated by suppression of the NF-κB pathway, primarily through selective downregulation of CXCL-8, as confirmed by both mRNA and protein analyses. Consistent with this mechanism, Ninj1 silencing also alleviated ox-LDL-induced endothelial apoptosis and dysfunction, suggesting that suppression of the NF-κB/CXCL-8 axis directly contributes to the observed functional protection. Interestingly, other NF-κB–regulated genes such as TNFAIP3 and CXCL-1 were not significantly affected, suggesting that Ninj1 fine-tunes inflammatory responses through a selective mechanism rather than broad NF-κB suppression. To further substantiate the mechanistic link between Ninj1 and the NF-κB/CXCL-8 pathway, we performed a rescue experiment by overexpressing CXCL-8 in Ninj1-silenced HUVECs. CXCL-8 overexpression restored the phosphorylation of NF-κB p65 that had been suppressed by Ninj1 knockdown, confirming that CXCL-8 acts as a downstream effector and a positive feedback mediator in the Ninj1/NF-κB signaling axis. These results suggest that Ninj1 promotes endothelial inflammation partly through CXCL-8-driven reinforcement of NF-κB activity, providing direct functional evidence for the causal involvement of this pathway in endothelial dysfunction. CXCL-8 is a potent pro-inflammatory chemokine known for its role in neutrophil recruitment and vascular inflammation (20). Beyond its chemotactic function, CXCL-8 can activate endothelial cells through autocrine signaling via CXCR1/2 receptors, forming a feedforward loop that amplifies NF-κB activity (21, 22). Our findings are consistent with prior studies demonstrating that blockade of the CXCL-8/CXCR2 axis limits monocyte transmigration and lesion development in early atherosclerosis (23). Thus, Ninj1 may serve as an upstream modulator of this pathway, offering a novel node of therapeutic intervention.

It is worth noting that mice lack a direct ortholog of human CXCL8. Instead, CXCL-1 and CXCL-2 serve as functional homologues that mediate neutrophil recruitment through the CXCR2 receptor and are transcriptionally regulated by NF-κB (24). Accordingly, while CXCL8 was assessed in human HUVECs, our *in vivo* analyses focused on murine Cxcl1 and Cxcl2 expression. Consistent with our *in vitro* findings, mPN12 treatment significantly reduced aortic Cxcl2 expression, while Cxcl1 showed a downward trend without statistical significance and Tnfaip3 remained unchanged. These results support the notion that Ninj1 regulates NF-κB–dependent chemokine signaling in a conserved manner across species. Moreover, previous studies have shown that NF-κB promotes the transcription of CXC chemokines, such as CXCL-8 and CXCL-1/CXCL-2, further reinforcing our proposed NINJ1–NF-κB–CXC chemokine axis as a unifying inflammatory mechanism (25, 26).

Importantly, our results challenge earlier conclusions that downplayed the relevance of endothelial Ninj1 in atherogenesis. Unlike previous studies that focused on macrophage-mediated inflammation (27, 28), our work highlights a distinct endothelial role for Ninj1. This apparent discrepancy likely stems from fundamental methodological differences: genetic knockout approaches may trigger compensatory mechanisms, whereas our pharmacological and gene-silencing approaches allow specific targeting of endothelial function. Our findings align with reports of Ninj1-mediated endothelial dysfunction in multiple sclerosis and diabetes, supporting the concept of context-dependent Ninj1 function across diseases and cellular environments (29). Furthermore, different Ninj1 isoforms (membrane-bound versus soluble) may exert distinct functions. Thus, our work does not contradict but rather expands the understanding of Ninj1’s cell type-specific roles. Therapeutically, targeting the endothelial Ninj1/NF-κB pathway could offer localized anti-inflammatory effects without the systemic immunosuppression of broad NF-κB inhibitors, potentially preserving beneficial Ninj1 functions in macrophages.

Our *in vivo* experiments further support the therapeutic potential of this strategy. Administration of the Ninj1 inhibitory peptide mPN12, which competitively disrupts Ninj1’s adhesion domain (18), significantly reduced plaque burden and lipid accumulation while preserving collagen content within lesions. The maintenance of collagen levels suggests that plaque stability is not compromised, a major concern in anti-inflammatory therapy (30). Moreover, this selective inhibition could preserve beneficial functions of soluble Ninj1 isoforms, avoiding the adverse outcomes observed with global Ninj1 deletion.

Despite these promising findings, our study has several limitations that warrant further investigation. First, endothelial-specific Ninj1 knockout models are required to delineate its *in vivo* contribution to atherosclerosis. Second, while our *in vivo* histological analyses mainly focused on plaque size and composition, the lack of endothelial-specific immunolabeling and immune infiltrate quantification limits the direct evidence for endothelial activation. Future studies employing endothelial markers (e.g., CD31, VCAM-1, ICAM-1), immune cell markers (e.g., CD68, CD3), as well as human aortic or coronary endothelial cells will be essential to directly validate the endothelial-specific role of Ninj1 under pathophysiological conditions relevant to atherosclerosis. Third, our *in vitro* experiments were performed in HUVECs, which, although widely used as a standard endothelial model, are of venous origin and may not fully represent arterial endothelial phenotypes. Moreover, in our *in vitro* setting, NINJ1 silencing was performed in unstimulated HUVECs to specifically dissect its cell-autonomous molecular role. Future studies will incorporate pro-inflammatory preconditioning (e.g., ox-LDL or TNF-α exposure) prior to silencing to better mimic the pathological endothelial state observed in atherosclerosis. Finally, while our rescue experiment confirmed reactivation of the CXCL-8/NF-κB cascade, corresponding functional rescue on endothelial apoptosis and adhesion remains to be explored, which will be a focus of future work.

**Figure 6:**
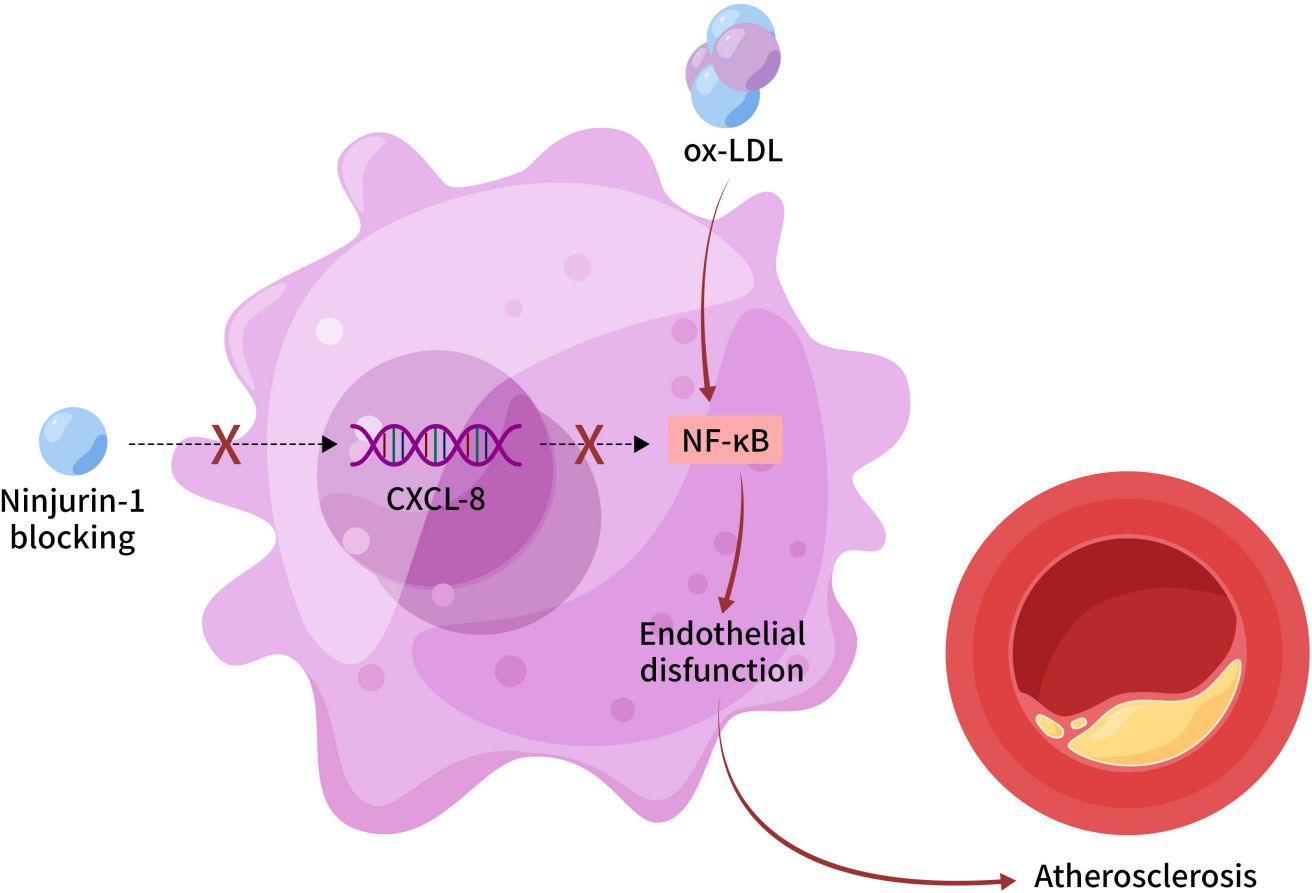
Proposed mechanism of endothelial Ninj1 in atherosclerosis pathogenesis. Ninjurin-1 in endothelial cells activates NF-κB signaling through upregulation of CXCL-8, leading to enhanced endothelial inflammation, dysfunction, and plaque progression. Pharmacological inhibition of Ninj1 suppresses this pathway and ameliorates vascular inflammation. This schematic represents a proposed mechanism based on the current *in vitro* and *in vivo* findings.

In summary, our study establishes a novel endothelial-specific mechanism by which Ninj1 drives atherosclerosis through CXCL-8-mediated NF-κB activation (**Figure 6**). By contributing to endothelial dysfunction and vascular inflammation, Ninj1 emerges as a promising, targetable mediator of early atherogenesis. These findings provide a strong foundation for the development of Ninj1-targeted therapies with improved safety and specificity for the treatment of atherosclerosis.

## Data Availability

The original contributions presented in the study are included in the article/**Supplementary Material**. Further inquiries can be directed to the corresponding authors.
