# Supplementary material for: Ninjurin-1 drives atherosclerosis progression via NF-κB/CXCL-8 activation in endothelial cells
Source: Front Immunol. 2025 Nov 7;16:1676216. doi: 10.3389/fimmu.2025.1676216 (PMC12634354; doi:10.3389/fimmu.2025.1676216)
Supplement: Supplementary file 1 [file DataSheet1.docx]

**Supplementary Table S1. Design of human Ninjurin-1 (NINJ1) shRNA sequences**

| **Vector** | **Target Name** | **Sequence (5'→3')** |
| --- | --- | --- |
| LV3(H1/GFP&Puro) | Human NINJ1 |  |
|  | NINJ1-Homo-47 (shNINJ1#1) | ACCGAGGAGTACGAGCTCAAC |
|  | NINJ1-Homo-318 (shNINJ1#2) | GGGTGCTGCTCATCTTCCTTG |
|  | NINJ1-Homo-422 (shNINJ1#3) | GTGGTAGTCAACATCTTCATC |
|  | NINJ1-Homo-1046 (shNINJ1#4) | GGCTCTGGTCTTAAGTGCCTG |

**Supplementary Table S2. Primer Sequences for RT-qPCR Analysis in Human and Mouse Genes**

| Gene Name | Species | Direction | Primer Sequence (5'→3') |
| --- | --- | --- | --- |
| Ninj1 | Human | Forward | CCTGGAAGACCTGGTCAATG |
|  |  | Reverse | GCTGGTAGAGGTGCTGGAAG |
| CXCL1 | Human | Forward | AGGCAGGGGAATGTATGTGC |
|  |  | Reverse | AAGCCCCTTTGTTCTAAGCCA |
| CXCL8 | Human | Forward | ACTGAGAGTGATTGAGAGTGGAC |
|  |  | Reverse | AACCCTCTGCACCCAGTTTTC |
| TNFAIP3 | Human | Forward | CAGCAGATTCAGGGCAAACA |
|  |  | Reverse | TCACAGGGCTCTGGAATAGC |
| GAPDH | Human | Forward | GGAGCGAGATCCCTCCAAAAT |
|  |  | Reverse | GGCTGTTGTCATACTTCTCATGG |
| CXCL1 | Mouse | Forward | ACTGCACCCAAACCGAAGTC |
|  |  | Reverse | TGGGGACACCTTTTAGCATCTT |
| CXCL2 | Mouse | Forward | CATCCAGAGCTTGAGTGTGACG |
|  |  | Reverse | GGCTTCAGGGTCAAGGCAAACT |
| TNFAIP3 | Mouse | Forward | TGTGGGGTGTTCAGGATACTG |
|  |  | Reverse | GTTCCGAGTGTCTGTCTCCTTA |
| GAPDH | Mouse | Forward | AGGTCGGTGTGAACGGATTTG |
|  |  | Reverse | TGTAGACCATGTAGTTGAGGTCA |


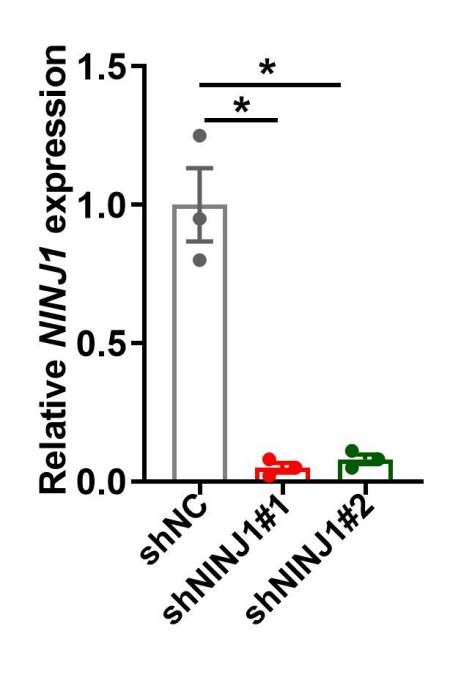


**Supplementary Figure S1 Validation of NINJ1 knockdown efficiency by RT-qPCR. High-Efficiency Lentiviral Transduction in HUVECs**


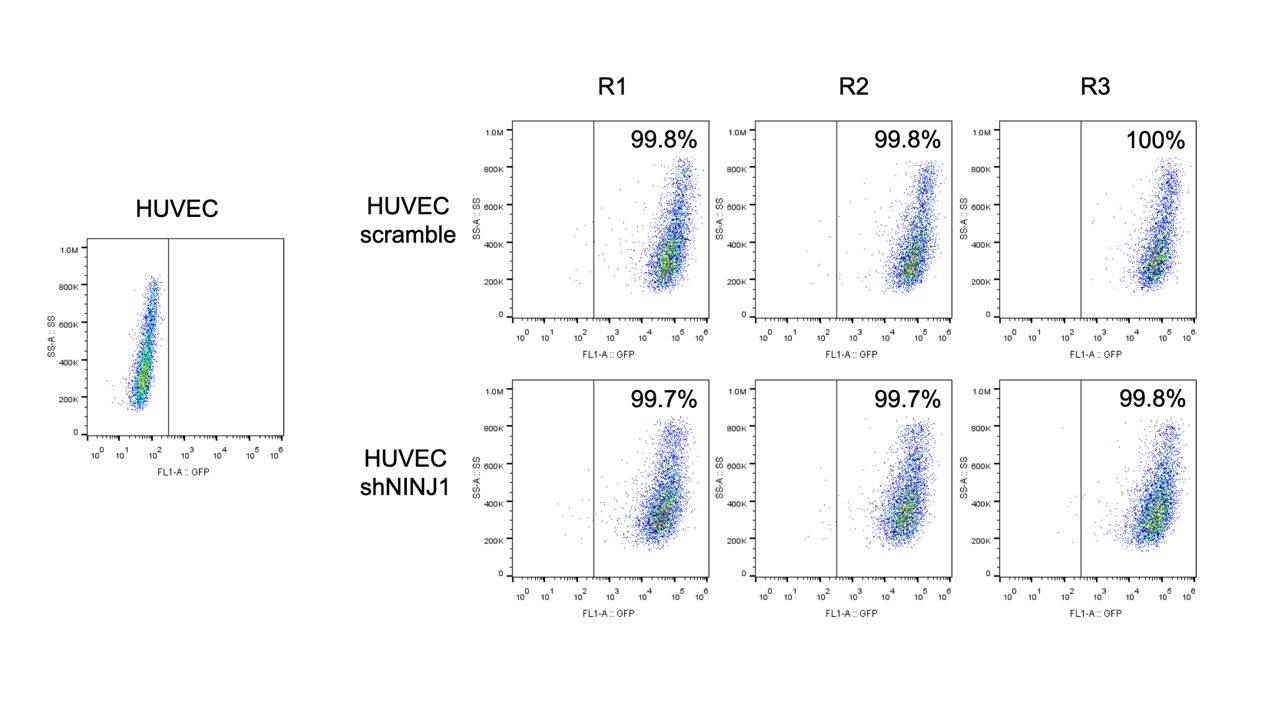


Figure S2. The transduction efficiency of the lentivirus in HUVECs was >99.5% based on GFP positivity assessed by flow cytometry.


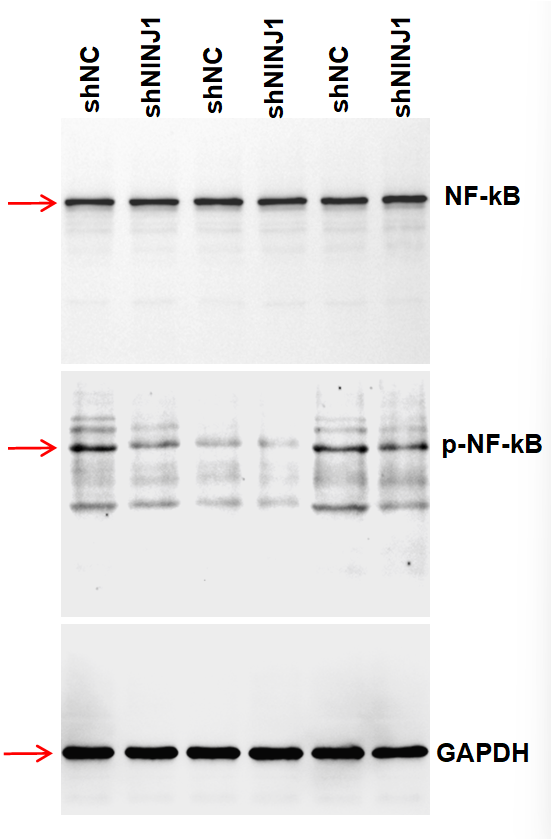


Supplementary Figure 3 The uncropped original membrane image showing sequential detection of p-p65
